# A Diagnostic Stewardship Intervention to Improve Utilization of 1,3 β-D-Glucan Testing at a Single Academic Center: Five-Year Experience

**DOI:** 10.1093/ofid/ofae358

**Published:** 2024-07-01

**Authors:** Jordan D Colson, Jonathan A Kendall, Takeru Yamamoto, Masako Mizusawa

**Affiliations:** Department of Pathology and Laboratory Medicine, University of Miami Miller School of Medicine, Miami, Florida, USA; Department of Internal Medicine, University of Missouri Kansas City, Kansas City, Missouri, USA; Department of Infectious Diseases, Kameda Medical Center, Kamogawa, Chiba, Japan; Section of Infectious Diseases, Department of Internal Medicine, University of Missouri Kansas City, Kansas City, Missouri, USA; Department of Pathology and Laboratory Medicine, Rutgers University Robert Wood Johnson Medical School, New Brunswick, New Jersey, USA; Department of Pathology and Laboratory Medicine, Monmouth Medical Center, Long Branch, New Jersey, USA

**Keywords:** biomarker, diagnostic stewardship, invasive fungal infections, test utilization, β-D-glucan

## Abstract

**Background:**

(1,3)- β-D-glucan (BDG) testing is one of the noninvasive tests to aid diagnosis of invasive fungal infections (IFIs). The study results have been heterogenous, and diagnostic performance varies depending on the risks for IFI. Thus, it is important to select appropriate patients for BDG testing to prevent false-positive results. An algorithmic diagnostic stewardship intervention was instituted at a single academic medical center to improve BDG test utilization.

**Methods:**

The BDG test order in the electronic health record was replaced with the BDG test request order, which required approval to process the actual test order. The approval criteria were (1) immunocompromised or intensive care unit patient and (2) on empiric antifungal therapy, or inability to undergo invasive diagnostic procedures. A retrospective observational study was conducted to evaluate the efficacy of the intervention by comparing the number of BDG tests performed between 1 year pre- and post-intervention. Safety was assessed by chart review of the patients for whom BDG test requests were deemed inappropriate and rejected.

**Results:**

The number of BDG tests performed per year decreased by 85% from 156 in the pre-intervention period to 24 in the post-intervention period. The average monthly number of BDG tests performed was significantly lower between those periods (*P* = .002). There was no delay in IFI diagnosis or IFI-related deaths in the patients whose BDG test requests were rejected. The sustained effectiveness of the intervention was observed for 5 years.

**Conclusions:**

Institution of the diagnostic stewardship intervention successfully and safely improved BDG test utilization.

Invasive fungal infections (IFIs) remain life-threatening diseases with high mortality despite advances in diagnostic tests and antifungal therapies [1]. Definitive diagnosis of IFI relies on histological and microbiological methods, which often require invasive procedures to obtain optimal samples [2]. Those who are most vulnerable to IFI are severely immunocompromised and/or critically ill in intensive care units (ICUs) [3], and they may not be amenable to invasive procedures due to increased risk of complications. Therefore, serological tests for fungal pathogens are often ordered as noninvasive diagnostic tests. (1,3)-β-D-glucan (BDG) is a cell wall polysaccharide found in most fungi, and detection of BDG has been utilized as a broad screening test for early detection of IFI [4]. While several BDG assays are available from different manufactures, Fungitell and Fungitell STAT (Associates of Cape Cod, East Falmouth, MA, USA) are currently the only assays cleared by Food and Drug Administration (FDA) in the United States. The diagnostic performance of BDG assays has been evaluated only for patients at risk for IFI who have high pretest probability, such as overtly immunocompromised patients with hematological malignancy, HIV, and a history of transplant, as well as ICU patients [5]. Thus, it is critically important to select the appropriate patients for BDG testing to minimize false-positive results, which may result in unnecessary antifungal therapy and additional diagnostic tests as those can cause adverse drug reactions, prolonged hospitalization, and increased cost of patient care. BDG belongs to a large group of polysaccharides found both throughout nature in bacteria, plants, algae, and fungi and in commercial products derived from those [6]. False positivity has been extensively described due to a variety of cross-reactions with sources as diverse as endotoxin, hemodialysis membranes, recent surgery, surgical gauze, recent transfusion, and various antibiotics [7]. False negativity occurs with the fungi that have no or little BDG in their cell walls such as *Blastomyces dermatitidis* in infective yeast form, *Cryptococcus* species, and *Mucorales* species [8]. False negativity is also not uncommon with the fungi that have abundant BDG in their cell walls such as *Aspergillus* spp. Angebault et al. found that BDG was undetectable in 48% of patients with proven and probable invasive aspergillosis at the time of diagnosis [9]. BDG tests have also been found to be less sensitive for *Candida parapsilosis* [10] and *C. auris* [11, 12] compared with other *Candida* species. While the literature is heterogeneous, a fair number of meta-analyses, predominantly including patients with malignancies, have evaluated the diagnostic accuracy of BDG tests for IFI and shown variable performance, with pooled sensitivities ranging from 61% to 95% and pooled specificities ranging from 63% to 90% [13–21]. For ICU patients, the studies on BDG testing have focused on antifungal stewardship and demonstrated varying degrees of success, but most studies have reported a negative predictive value (NPV) >90% [5]. The European Organisation for Research and Treatment of Cancer and Mycoses Study Group conducted a systematic review to assess the role of BDG testing for diagnosis of IFI in adults and suggested that a negative BDG test result can be used to withhold or discontinue empirical antifungal therapy for ICU patients as NPVs are generally high [5]. The use of this BDG-driven antifungal stewardship strategy is supported by the data from 2 randomized trials in ICU patients [22, 23]. Considering the available evidence described above, a medium-strength, algorithm-based intervention was implemented at a single academic center to optimize utilization of BDG testing.

## METHODS

The test request approval process for BDG testing was introduced on November 28, 2018, at University Health Truman Medical Center in Kansas City, Missouri. Truman Medical Center is a 2-campus acute care academic teaching hospital with a level 1 trauma center. The total number of beds is 348, including 34 ICU beds (14 for medical, 10 for surgical, and 10 for cardiac ICUs). Comprehensive subspecialty services including hematology/oncology service and HIV care are offered for adult patients. However, hematopoietic stem cell and organ transplant services are not available. There is no designated hematology/oncology unit or bed, but hematology/oncology patients were preferably admitted to a specific medical/surgical unit with 18 beds. Pediatric patient care is limited to outpatient family medicine practice and adolescent primary care services only. The proportion of admissions due to advanced HIV (defined as CD4 <200 cells/mm^3^) at our institution was 0.5% of all hospitalizations during the study period. More than 90% of the IFIs at our institution are candidemia, and the most common species is *Candida albicans,* followed by *C. glabrata* and *C. parapsilosis*. The BDG test is not performed by the laboratory in the hospital, and the specimens are sent out to a reference laboratory during business hours on weekdays only. The Fungitell assay (Associates of Cape Cod, Inc., East Falmouth, MA, USA) was used for BDG testing at the reference laboratory (Eurofins Viracor, Lee's Summit, MO, USA). The assay range at the reference laboratory was 31–500 pg/mL for serum samples and 45–500 pg/mL for bronchoalveolar lavage (BAL) samples. The cutoff value for positivity was ≥80 pg/mL for serum samples. There is no established cutoff for BAL BDG positivity, and the reference laboratory reported BAL BDG values only with no interpretations. Diagnostic performance of the BAL BDG for IFI was found to be poor in a meta-analysis of 6 BAL BDG studies, including 838 patients who showed a pooled sensitivity of 52% and a pooled specificity of 56% using the cutoff value of 80 pg/mL in 4 studies, 100 pg/mL in 1 study, and 107 pg/mL in 1 study [24]. However, higher sensitivity was reported in patients with *Pneumocystis jirovecii* pneumonia, with a median BAL BDG level of >500 pg/mL [25]. Using a cutoff of 783 pg/mL for the BDG values from 100 BAL samples, 1 study showed a sensitivity of 72% and a specificity of 71% for diagnosis of *Pneumocystis jirovecii* pneumonia (PJP) [26]. Based on the available evidence and our own experience, a BAL BDG >500 pg/mL was considered positive at our institution and was used to aid diagnosing PJP as a local practice. The direct cost of BDG testing was US$180 at the beginning of this study and remained the same until July 2022, when the direct cost was increased to US$193.

### Approval Process

The preexisting BDG test order was replaced with a BDG test request order in the electronic health records (EHRs). When a specimen for the BDG test was collected and received, a test request order appeared on the task queue for the pathology residents, and the BDG test request appeared as a pending test in the laboratory result screen in the patient EHR. An on-call pathology resident performed the chart review first and then called the ordering provider to obtain additional information and directly discuss the case. When an approval/rejection decision was not straightforward based on the test approval algorithm, the on-call resident contacted the microbiology laboratory director for discussion before decision-making.

BDG test requests were approved based on the following criteria:

- The patient is hospitalized in the ICU and is currently on empiric antifungal therapy for suspected IFI, but if the BDG test is negative, the antifungal therapy will be discontinued.
- The patient has an immunocompromised condition, defined as neutropenia, HIV, malignancy, history of stem cell transplant, history of solid organ transplant, or being on immunosuppressive therapy, and is suspected to have pulmonary invasive aspergillosis but cannot undergo bronchoscopy.
- The patient has an immunocompromised condition as defined above and is suspected to have *Pneumocystis jirovecii* pneumonia but cannot undergo bronchoscopy.
- The patient has an immunocompromised condition as defined above and was started on empiric antifungal therapy for suspected *Pneumocystis jirovecii* pneumonia before bronchoscopy.
- The patient has an immunocompromised condition and was started on empiric antifungal therapy for suspected candidemia.

**Figure 1.**
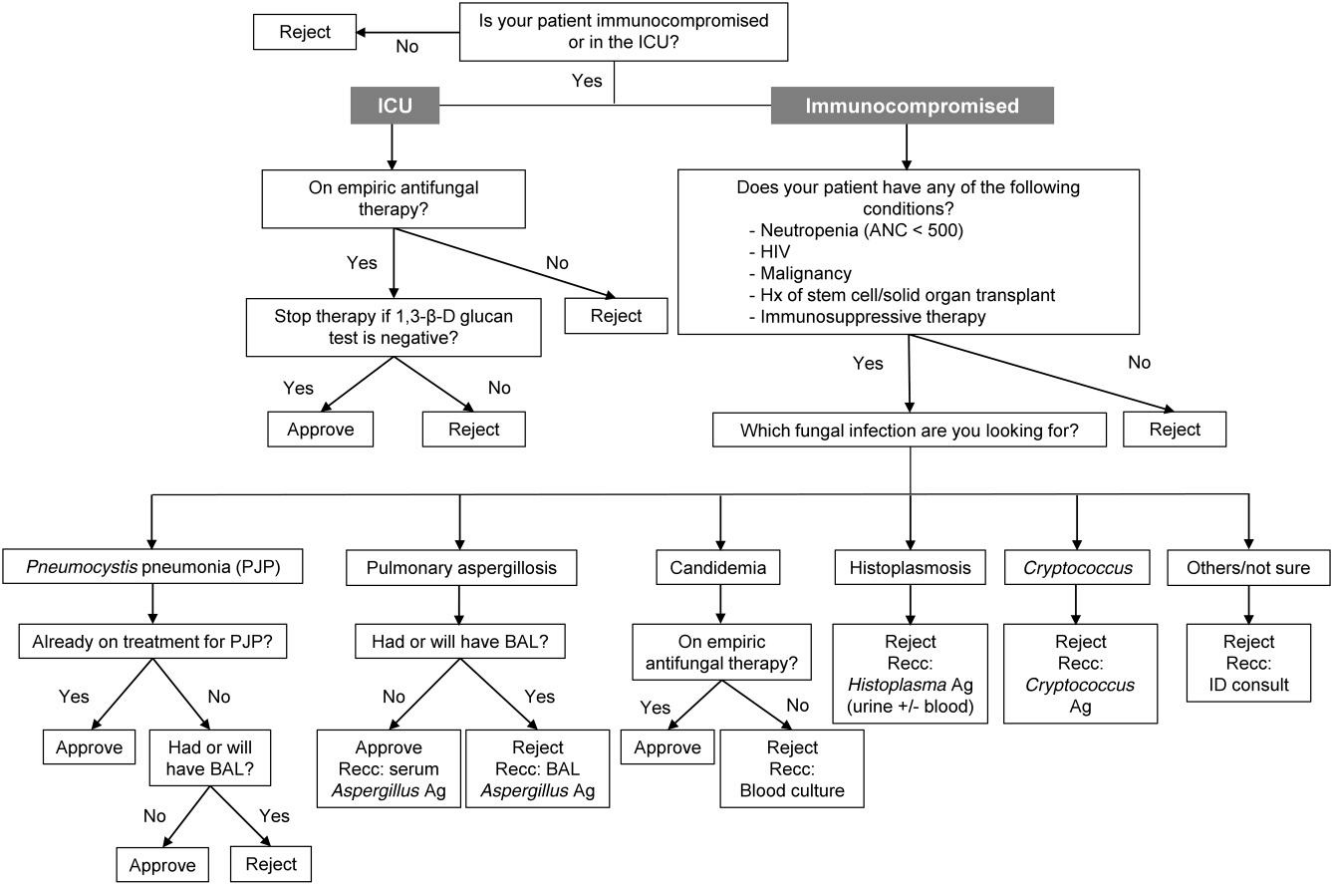
The approval algorithm for the 1,3-β-D-glucan test request. Abbreviations: Ag, antigen; ANC, absolute neutrophil count; BAL, bronchoalveolar lavage; ICU, intensive care unit; PJP, *Pneumocystis jirovecii* pneumonia; Recc, recommend.

Additional diagnostic tests and infectious disease consultation were recommended based on what fungal infection the ordering provider was in search of. The details of the test approval algorithm are shown in Figure 1. For a patient who was suspected to have PJP, Grocott's methenamine silver (GMS) stain was routinely performed on BAL, and *Pneumocystis jirovecii* polymerase chain reaction (PCR) was also available to order on BAL. When the patient was not on empiric treatment for PJP and was undergoing or already had BAL, the BDG test request was rejected because BAL GMS stain and *Pneumocystis jirovecii* PCR are more sensitive and specific for diagnosis of PJP compared with BDG testing. However, if the patient had been on empiric treatment for PJP, the BDG test request was approved regardless of a plan for bronchoscopy because empiric treatment could decrease the sensitivities of GMS stain and *Pneumocystis jirovecii* PCR on BAL [27] and BDG testing might provide additional diagnostic value.

The approval/rejection status of the test request was shown in the laboratory information system (LIS) as well as in the patient EHR once the decision was made on the pathology resident task queue. Cerner platforms (Cerner Corporation, St Louis, MO, USA) were used for both the LIS and EHR. The decision on the test approval/rejection was directly communicated with the ordering provider and the send-out section in the laboratory as soon as possible. The BDG test results of the ICU patients who were empirically on antifungal therapy were monitored by the antimicrobial stewardship program. If the BDG test of the ICU patient was negative but the patient was still on empiric antifungal therapy, the infectious disease pharmacist reached out to the ICU team and discussed discontinuation of antifungal agents.

### Data Collection and Outcomes

Following approval from the institutional review board, a retrospective EHR review was conducted for the patients who had BDG tests during the year before the intervention (pre-intervention group) and those who had BDG test requests during the year after the intervention (post-intervention group). The post-intervention group was further divided into the approved BDG test group and the rejected BDG test group. The patient and order characteristics were compared between the pre- and post-intervention groups as well as between the approved and rejected BDG test groups. The primary outcome was change in the average monthly number of BDG tests performed from the pre-intervention period to the post-intervention period. To ensure the safety of the intervention, IFI-related deaths and delayed IFI diagnoses in the rejected BDG group were monitored, and the average test turnaround time and test positivity rate were compared between the pre- and post-intervention groups as the secondary outcomes. As a part of quality assurance practice, we continued to monitor the numbers of the BDG test requests and BDG tests performed beyond the first year of the post-intervention period.

### Statistical Analyses

Categorical data were expressed as counts and percentages. Continuous data were expressed as means and standard deviations or medians and interquartile ranges, as appropriate. Categorical variables were compared using the χ^2^ test or Fisher exact test, and continuous variables were compared using the *t* test or Wilcoxon rank-sum test, as appropriate.

## RESULTS

**Figure 2.**
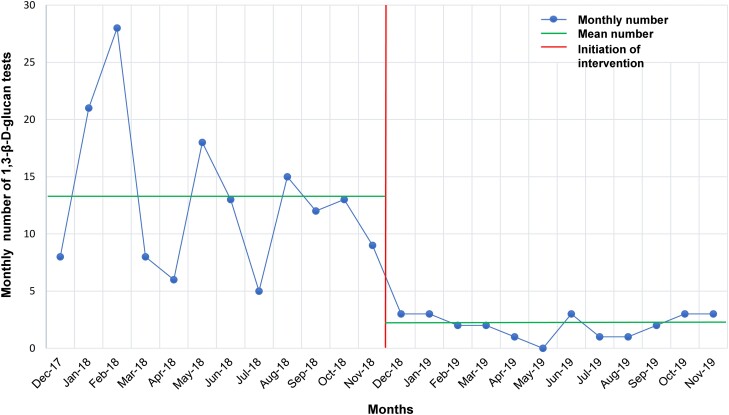
Monthly numbers of 1,3-β-D-glucan tests performed in the pre-intervention period and approved 1,3-β-D-glucan tests in the post-intervention period.

The number of BDG tests performed per year decreased by 85% from 156 in the pre-intervention period to 24 in the first year of the post-intervention period. The number of BDG test requests in the first year of the post-intervention period was 65, and 41 of them were rejected based on the test approval criteria. The most common reason for the rejection was that the patient was not immunocompromised and not in the ICU (72%). Other reasons for rejection are described in Supplementary Table 1. The average monthly numbers of BDG tests performed were significantly lower in the first year of the post-intervention period compared with the pre-intervention period (*P* = .002) (Figure 2). This significant reduction was maintained after adjusting the monthly numbers of BDG tests by 1000 patient-days for inpatients (*P* = .002) (Supplementary Figure 1). The average monthly number of BDG test request orders was also significantly lower in the first year of the post-intervention period compared with that of BDG test orders in the pre-intervention period (*P* = .004) (Supplementary Figure 2). Compared with the pre-intervention period, during which all 156 tests ordered were performed, the direct cost of BDG tests was US$23 760 lower during the first year of the post-intervention period, during which 41 of the 65 tests ordered were rejected and only 24 tests were performed.

The pre-intervention group had slightly more male patients and more frequent diagnostic work-up tests (complete blood count within 48 hours and chest imaging) compared with the post-intervention BDG test request group, but otherwise there were no significant differences in the parameters (Table 1). The providers were ordering BDG tests because of leukocytosis and radiographic evidence of infections in the pre-intervention group more frequently compared with the post-intervention BDG test request group (Table 2). Within the post-intervention group, the approved test group was more likely to have fever within 48 hours, consolidation/pneumonia on chest imaging, and infectious disease (ID) physician's agreement on BDG testing compared with the rejected test group (Table 3). As expected, the proportion of those who were on empiric antifungal therapy was higher in the approved test group as being on antifungal therapy was one of the test approval criteria. The frequency of ordering BDG tests due to leukocytosis and radiographic evidence of infections was higher in the approved test group compared with the rejected test group (Table 4). The test positivity rate was significantly higher in the post-intervention group compared with the pre-intervention group (45.8% vs 25.3%; *P* = .038) (Supplementary Table 2). There was no significant difference in the average test turnaround time between the pre- and post-intervention groups (49.4 hours vs 46.9 hours; *P* = .73) (Supplementary Table 2). There was also no significant difference in the average number of days of therapy (DOT)/1000 patient-days for all antifungal agents between the pre-intervention period and the first year of the post-intervention period (19.67 vs 16.61; *P* = .06).

**Table 1.** Comparison of Patient Characteristics Between 1,3-β-D-Glucan Test Orders in the Pre-intervention Group and Order Requests in the Post-intervention Group

	Pre-intervention 1,3-β-D-Glucan Test Orders (n = 156)	Post-intervention 1,3-β-D-Glucan Test Requests (n = 65)	*P* Value
**Patient characteristics**			
Age, mean [SD], y	52.5 [14.0]	52.3 [13.8]	.96
Male, No. (%)	106 (67.9)	33 (50.8)	**.016**
**Immunocompromised condition**,^a^ **No. (%)**	52 (33.3)	26 (40.0)	.35
Neutropenia	6 (3.8)	4 (6.2)	.49
HIV	29 (18.6)	12 (18.5)	.98
Hematologic malignancy	8 (5.1)	5 (7.7)	.48
Organ transplant	2 (1.3)	1 (1.5)	1.0
Immunosuppression for autoimmune disease	6 (3.8)	3 (4.6)	.68
Chemotherapy for malignancy	14 (9.0)	9 (13.8)	.28
Primary immunodeficiency	1 (0.6)	0 (0)	1.0
**Clinical and laboratory findings,** ^a^ **No. (%)**			
Fever within 48 h, No. (%)	41 (26.3)	24 (36.9)	.11
CBC tested within 48 h, No. (%)	140 (89.7)	45 (69.2)	**.0017**
Leukocytosis (WBC >12 × 10^3^ cells/μL)	50 (32.1)	27 (41.5)	.18
Leukopenia (WBC <4 × 10^3^ cells/μL)	20 (12.8)	12 (18.5)	.28
**Chest imaging, No. (%)**			
Chest x-ray within 30 d	130 (83.3)	49 (75.4)	.17
CT chest within 30 d	123 (78.8)	47 (72.3)	.29
Normal radiographs	4 (25.6)	3 (4.6)	.42
No chest imaging within 30 d	0 (0)	4 (6.2)	**.0070**
**Radiographic abnormalities,** ^a^ **No. (%)**			
Nodule^b^	48 (30.8)	15 (23.1)	.25
Cavitation	26 (16.7)	4 (6.2)	.051
Mass	14 (9.0)	2 (3.1)	.16
Consolidation/pneumonia	37 (23.7)	10 (15.4)	.17
Opacity	95 (60.9)	39 (60.0)	.90
**ID consult within 24 h of order, No. (%)**	63 (40.4)	33 (50.8)	.16
Recommend/agree with testing	11 (7.1)	7 (10.8)	.36
**Empiric antifungal therapy administration**	35 (22.4)	16 (24.6)	.72
**Duration of empiric antifungal therapy, No. of d [SD]**	3.9 [2.3]	3.5 [2.2]	.61
**Mortality and readmission, No. (%)**			
All-cause 30-d death	22 (14.1)	6 (9.2)	.32
IFI-related 30-d death	1 (0.6)	0 (0)	1.0
All-cause 30-d readmission	13 (8.3)	11 (16.9)	.062

Statistically significant *P* values are highlighted in bold. Abbreviations: CBC, complete blood count ; CT, computed tomography; ID, infectious diseases; IFI, invasive fungal infection.

^a^Not mutually exclusive.

^b^Including granulomas, calcification, spiculated and tree-in-bud findings.

**Table 2.** Comparison of Test Order Characteristics Between 1,3-β-D-Glucan Test Orders in the Pre-intervention Group and Order Requests in the Post-intervention Group

	Pre-intervention 1,3-β-D-Glucan Test Orders (n = 156)	Post-intervention 1,3-β-D-Glucan Test Requests (n = 65)	*P* Value
**Service type, No. (%)**			
Internal medicine/primary care	40 (25.6)	21 (32.3)	.31
Pulmonology	92 (59.0)	31 (47.7)	.12
Surgery	21 (13.5)	10 (15.4)	.71
Infectious disease	3 (1.9)	2 (3.1)	.63
Others	0 (.0)	1 (1.5)	.29
**Location, No. (%)**			.098
Outpatient	21 (13.5)	13 (20.0)	
Inpatient non-ICU	82 (52.6)	24 (36.9)	
ICU	53 (34.0)	28 (43.1)	
**Ordering provider type, No. (%)**			.25^a^
Resident	80 (51.3)	44 (67.7)	
Fellow	58 (37.2)	15 (23.1)	
Attending	17 (10.9)	6 (9.2)	
Nurse practitioner	1 (0.6)	0 (0)	
**Specimen type, No. (%)**			
Serum	111 (71.2)	50 (76.9)	.38
Bronchoalveolar lavage	45 (28.8)	15 (23.1)	.38
**Reason for ordering,** ^b^ **No. (%)**			
Fever	12 (7.7)	3 (4.6)	.56
Leukocytosis	22 (14.1)	1 (1.5)	**.0033**
Radiographic evidence of lung infection	110 (70.5)	31 (47.7)	**.0013**
Hypoxemic respiratory failure	18 (11.5)	3 (4.6)	.14
Sepsis or septic shock	25 (16.0)	17 (26.2)	.080
Fungal infection suspected	19 (12.2)	7 (10.8)	.77

Statistically significant *P* values are highlighted in bold. Abbreviation: ICU, intensive care unit.

^a^Trainees (resident/fellow) vs. independent practitioners (attending/nurse practitioner) were compared.

^b^Not mutually exclusive.

**Table 3.** Comparison of Patient Characteristics Between Approved and Rejected 1,3-β-D-Glucan Test Requests in the Post-intervention Group

	1,3-β-D-Glucan Test Requests Approved (n = 24)	1,3-β-D-Glucan Test Requests Rejected (n = 41)	*P* Value
**Patient characteristics**			
Age, mean [SD], y	54.2 [14.4]	51.2 [13.6]	.42
Male, No. (%)	17 (70.8)	16 (39.0)	**.013**
**Immunocompromised condition,** ^a^ **No. (%)**	12 (50.0)	14 (34.1)	.21
Neutropenia	2 (8.3)	2 (4.9)	.62
HIV	6 (25.0)	6 (14.6)	.30
Hematologic malignancy	3 (12.5)	2 (4.9)	.35
Organ transplant	1 (4.2)	0 (0)	.37
Immunosuppression for autoimmune disease	1 (4.2)	2 (4.9)	1.0
Chemotherapy for malignancy	3 (12.5)	6 (14.6)	1.0
**Clinical and laboratory findings, ^a^ No. (%)**			
Fever within 48 h, No. (%)	15 (62.5)	9 (22.0)	**.001**
CBC within 48 h, No. (%)	19 (79.2)	26 (63.4)	.18
Leukocytosis (WBC >12 × 10^3^ cells/mcL)	12 (50.0)	15 (36.6)	.29
Leukopenia (WBC <4 × 10^3^ cells/mcL)	3 (12.5)	9 (22.0)	.51
**Chest imaging, No. (%)**			
Chest x-ray within 30 d	21 (87.5)	28 (68.3)	.083
CT chest within 30 d	16 (66.7)	31 (75.6)	.43
Normal radiographs	1 (4.2)	2 (4.9)	1.0
No chest imaging within 30 d	1 (4.2)	3 (7.3)	1.0
**Radiographic abnormalities,** ^a^ **No. (%)**			
Nodule^b^	4 (16.7)	11 (26.8)	.54
Cavitation	1 (4.2)	3 (7.3)	1.0
Mass	1 (4.2)	1 (2.4)	1.0
Consolidation/pneumonia	7 (29.2)	3 (7.3)	**.031**
Opacity	17 (70.8)	22 (53.7)	.17
**ID consult within 24 h of order, No. (%)**	15 (62.5)	18 (43.9)	.15
Recommend/agree with testing	7 (29.2)	2 (4.9)	**.0010**
**Empiric antifungal therapy administration**	12 (50.0)	4 (9.8)	**.0010**
**Duration of empiric antifungal therapy, No. of d [SD]**	3.7 [1.8]	3.0 [3.7]	.60
**Mortality and readmission, No. (%)**			
All-cause 30-d death	2 (8.3)	4 (9.8)	1.0
IFI-related 30-d death	0 (0)	0 (0)	1.0
All-cause 30-d readmission	7 (29.2)	5 (12.2)	.089

Statistically significant *P* values are highlighted in bold. Abbreviations: CBC, complete blood count; CT, computed tomography; ID, infectious diseases; IFI, invasive fungal infection; WBC, white blood cell.

^a^Not mutually exclusive.

^b^Including granulomas, calcification, spiculated nodules, and tree-in-bud findings.

**Table 4.** Comparison of Order Characteristics Between Approved and Rejected 1,3-β-D-Glucan Test Requests in the Post-intervention Group

	1,3-β-D-Glucan Test Requests Approved (n = 24)	1,3-β-D-Glucan Test Requests Rejected (n = 41)	*P* Value
**Service type, No. (%)**			
Internal medicine/primary care	8 (33.3)	13 (31.7)	.892
Pulmonology	10 (41.7)	22 (53.7)	.351
Surgery	6 (25.0)	3 (7.3)	.066
Infectious disease	0 (0)	2 (4.9)	.527
Others	0 (0)	1 (2.4)	1.000
**Location, No. (%)**			
Outpatient	1 (4.2)	12 (29.3)	**.022**
Inpatient non-ICU	8 (33.3)	16 (39.0)	.646
ICU	15 (62.5)	13 (31.7)	**.016**
**Ordering provider type, No. (%)**			
Resident	20 (83.3)	24 (58.5)	**.039**
Fellow	2 (8.3)	13 (31.7)	**.036**
Attending	2 (8.3)	4 (9.8)	1.000
**Specimen type, No. (%)**			
Serum	22 (91.7)	28 (68.3)	**.036**
Bronchoalveolar lavage	2 (8.3)	13 (31.7)	**.036**
**Reason for order,** ^a^ **No. (%)**			
Fever	12 (7.7)	3 (4.6)	.562
Leukocytosis	22 (14.1)	1 (1.5)	**.003**
Radiographic evidence of lung infection	110 (70.5)	31 (47.7)	**.001**
Hypoxemic respiratory failure	18 (11.5)	3 (4.6)	.135
Sepsis or septic shock	25 (16.0)	17 (26.2)	.080
Fungal infection suspected	19 (12.2)	7 (10.8)	.767

Statistically significant *P* values are highlighted in bold. Abbreviation: ICU, intensive care unit.

^a^Not mutually exclusive.

A retrospective chart review of the patients whose BDG test requests were rejected (the rejected BDG group) revealed 2 cases of IFI. The detailed case descriptions are in the Supplementary Data. In both cases, the lack of BDG testing did not delay those diagnoses. Other diagnostic tests for IFI performed in the rejected test group are shown in Supplementary Table 3. The approved test group had 3 cases of IFI (3 PJP), and the pre-intervention group had 6 cases of IFI (1 disseminated histoplasmosis, 1 pulmonary invasive aspergillosis, 1 pulmonary coccidioidomycosis, and 3 PJP). In the pre-intervention group, there were 22 deaths (14.1%) within 30 days of BDG testing, 1 of which was IFI-related (disseminated histoplasmosis). In the post-intervention group, there were 6 deaths (9.2%) within 30 days of the time of BDG test request. There were no IFI-related deaths observed in either the rejected BDG test group or the approved BDG test group.

Continued quality assurance monitoring data demonstrated sustained reductions in the number of BDG tests for 5 years after the introduction of the intervention (Table 5).

**Table 5.** Number of 1,3-β-D-Glucan Test Requests, Number of Approved Tests, and Direct Cost Savings by Test Request Rejection

	Pre-intervention (11/28/2017–11/27/2018)	1st Year (11/28/2018–11/27/2019)	2nd Year (11/28/2019–11/27/2020)	3rd Year (11/28/2020–11/27/2021)	4th Year (11/28/2021–11/27/2022)	5th Year (11/28/2022–11/27/2023)
No. of test requests	156	65	49	43	68	81
No. of approved tests	156	24	22	12	27	21
Direct cost saved by test request rejection, US$	0	7380	4680	5580	7536	11 580

## DISCUSSION

Our diagnostic stewardship intervention successfully led to an 85% reduction in the number BDG tests performed without delay in diagnosis of IFI due to lack of BDG testing or IFI-related deaths during the first year post-intervention. We observed sustained effectiveness of the intervention for 5 years. Of note, the number of the orders placed by the providers to request BDG testing was 63% lower during the first year of the post-intervention period compared with the pre-intervention period, during which no diagnostic stewardship intervention existed for BDG testing. This observation suggests that the presence of order restriction itself might have affected the providers’ decisions on ordering and prevented some of the unnecessary BDG tests. Decreased numbers of BDG test requests during the second and the third years of the post-intervention period may be associated with the severe acute respiratory syndrome coronavirus 2 (SARS-CoV-2) pandemic, during which hospital admissions for non-SARS-CoV-2 diseases markedly declined. However, despite the increased numbers of BDG test request orders during the fourth and fifth years, the numbers of BDG tests performed were kept low by rejecting inappropriate test requests. The total direct cost saved by test request rejection was US$36 756 over 5 years.

One of the challenges in diagnostic stewardship for BDG testing would be difficulty in extracting a complete list of the patient's underlying diseases and risk factors for IFI through the EHR because the diagnostic codes entered may not be accurate or complete and risk factors for IFI may not be systematically documented in the physicians’ notes. Therefore, we included manual chart review and direct discussion with ordering providers in order to safely implement the intervention. Through direct discussion with ordering providers, we were also able to recommend ID consult and more specific diagnostic tests for IFI. It may be also challenging to have institutional agreement on the criteria for BDG test approval because of variabilities in clinical practice and different levels of understanding of test performance among ordering providers. We took local practice into consideration when we designed the BDG test approval algorithm in order to overcome resistance from ordering providers.

Fabre et al. investigated BDG testing among non-neutropenic adult inpatients at a single large academic center where BDG testing is unrestricted [28]. The authors found that BDG orders were inappropriate in 49% of cases due to lack of host risk factors or consistent clinical presentations, and up to 30% of orders were completely avoidable [28]. Based on those results, they included a list of indications for BDG in the EHR with a message stating that orders without appropriate indications will be audited but elected not to require pre-authorization because of existing demands on the ID consult and stewardship teams, in addition to the complexities of identifying the correct patient population for testing, which requires knowledge not held by microbiology technicians [28]. Ito et al. conducted a retrospective study to evaluate the appropriateness of fungal serologic tests performed for inpatients at a single large academic center using similar criteria (ie, host risk factors and consistent clinical presentations) and found that 74.8% of the BDG tests conducted were deemed inappropriate [29]. Need of guidance on patient selection to reduce inappropriate ordering was mentioned, but no specific intervention plan was described in their study [29]. Kritikos et al. reported a 26% appropriate usage rate of BDG in search of invasive candidiasis in the ICU in their study [30]. During the first study period, 57% of therapeutic decisions, primarily through the cessation of antifungal therapy, were impacted by a BDG result [30]. Before the second study period, they created a pocket card that includes indications for BDG testing and decision-support algorithms for discontinuation of empiric antifungal therapy and initiation of preemptive antifungal therapy guided by BDG test results [30]. The card was distributed to all ICU attending physicians and ID consultants, but adherence to the algorithm was low (26%), and no benefit of the intervention was observed during the second period [30]. Similar to these 3 studies, we found that a large proportion of BDG test orders were inappropriate and then went one step further to require ordering providers to obtain approval for BDG testing, which led to sustained reductions in the number of inappropriate BDG tests over 5 years.

Our study has several limitations. First, it was conducted at a single academic center with no hematopoietic stem cell or solid organ transplant center, and the test approval algorithm was designed based on local epidemiology and clinical practice within our institution. Therefore, the results may not be generalizable to other institutions with different epidemiology, patient populations, and clinical practice. Second, our intervention involved detailed chart review and direct discussion with ordering providers, which are time-consuming and may not be feasible at institutions where a large number of BDG tests are ordered. The feasibility of the intervention may also be impacted by EHR capacity and availability of ID consultation. Third, our test approval algorithm included being on empiric antifungal therapy in the approval criteria. Although our institution did not experience significant increases in empiric antifungal use, our criteria may have the potential to cause this unintended consequence at other institutions. Fourth, our laboratory does not perform BDG testing; the specimens are sent out to a reference laboratory during business hours on weekdays only. Order restriction may affect the BDG test turnaround time differently at institutions where BDG testing is performed by their own laboratories. However, we believe that similar diagnostic stewardship interventions for BDG testing would be feasible at other institutions by adjusting the approval algorithm based on local epidemiology, patient populations, and clinical practice and limiting the intervention to certain units or floors where inappropriate BDG tests are commonly ordered. A multidisciplinary approach involving the laboratory, ID, and antimicrobial stewardship teams is required for implementation of diagnostic stewardship and successful optimization of BDG test utilization.

In conclusion, the algorithm-based diagnostic stewardship intervention for BDG testing was implemented at a single academic center and effectively reduced the number of inappropriate BDG tests without delay in diagnosis of IFI or IFI-related deaths. Sustained effectiveness of the intervention was observed for 5 years. More data and experience in diagnostic stewardship interventions for BDG testing are needed to optimize BDG test utilization across diverse health care facilities to enhance patient care at a larger scale.

## Supplementary Material

ofae358_Supplementary_Data

## Supplementary Data

Supplementary materials are available at *Open Forum Infectious Diseases* online. Consisting of data provided by the authors to benefit the reader, the posted materials are not copyedited and are the sole responsibility of the authors, so questions or comments should be addressed to the corresponding author.

## References

[1] Brown GD, Denning DW, Gow NAR, Levitz SM, Netea MG, White TC. Hidden killers: human fungal infections. Sci Transl Med 2012; 4:165rv13. 10.1126/scitranslmed.3004404 23253612

[2] Riwes MM, Wingard JR. Diagnostic methods for invasive fungal diseases in patients with hematologic malignancies. Expert Rev Hematol 2012; 5:661–9. 23216596 10.1586/ehm.12.53 PMC3563387

[3] Rüping MJGT, Vehreschild JJ, Cornely OA. Patients at high risk of invasive fungal infections. Drugs 2008; 68:1941–62. 18778118 10.2165/00003495-200868140-00002

[4] Theel ES, Doern CD. β-d-glucan testing is important for diagnosis of invasive fungal infections. J Clin Microbiol 2013; 51:3478–83. 23850953 10.1128/JCM.01737-13 PMC3889722

[5] Lamoth F, Akan H, Andes D, et al Assessment of the role of 1,3-β-d-glucan testing for the diagnosis of invasive fungal infections in adults. Clin Infect Dis 2021; 72:S102–8. 33709130 10.1093/cid/ciaa1943

[6] Du B, Meenu M, Liu H, Xu B. A concise review on the molecular structure and function relationship of β-glucan. Int J Mol Sci 2019; 20:4032. 31426608 10.3390/ijms20164032 PMC6720260

[7] Finkelman MA . Specificity influences in (1→3)-β-d-glucan-supported diagnosis of invasive fungal disease. J Fungi (Basel) 2020; 7:14. 33383818 10.3390/jof7010014 PMC7824349

[8] Associates of Cape Cod, INC . Fungitell® Assay [Package Insert]. Associates of Cape Cod, Inc.; 2023.

[9] Angebault C, Lanternier F, Dalle F, et al Prospective evaluation of serum β-glucan testing in patients with probable or proven fungal diseases. Open Forum Infect Dis 2016; 3:ofw128. 27419189 10.1093/ofid/ofw128 PMC4942764

[10] Mikulska M, Giacobbe DR, Furfaro E, et al Lower sensitivity of serum (1,3)-β-d-glucan for the diagnosis of candidaemia due to *Candida parapsilosis*. Clin Microbiol Infect 2016; 22:646.e5–8. 10.1016/j.cmi.2016.05.020 27256062

[11] Farooqi J, Niamatullah H, Irfan S, Zafar A, Malik F, Jabeen K. Comparison of β-D-glucan levels between *Candida auris* and other *Candida* species at the time of candidaemia: a retrospective study. Clin Microbiol Infect 2021; 27:1519.e1–5. 10.1016/j.cmi.2021.05.031 34111581

[12] Mikulska M, Balletto E, Castagnola E, Mularoni A. Beta-D-glucan in patients with haematological malignancies. J Fungi (Basel) 2021; 7:1046. 34947028 10.3390/jof7121046 PMC8706797

[13] Lu Y, Chen Y-Q, Guo Y-L, Qin S-M, Wu C, Wang K. Diagnosis of invasive fungal disease using serum (1→3)-β-D-glucan: a bivariate meta-analysis. Intern Med 2011; 50:2783–91. 22082890 10.2169/internalmedicine.50.6175

[14] Karageorgopoulos DE, Vouloumanou EK, Ntziora F, Michalopoulos A, Rafailidis PI, Falagas ME. β-D-glucan assay for the diagnosis of invasive fungal infections: a meta-analysis. Clin Infect Dis 2011; 52:750–70. 21367728 10.1093/cid/ciq206

[15] Lamoth F, Cruciani M, Mengoli C, et al β-glucan antigenemia assay for the diagnosis of invasive fungal infections in patients with hematological malignancies: a systematic review and meta-analysis of cohort studies from the third European Conference on Infections in Leukemia (ECIL-3). Clin Infect Dis 2012; 54:633–43. 22198786 10.1093/cid/cir897

[16] Onishi A, Sugiyama D, Kogata Y, et al Diagnostic accuracy of serum 1,3-β-D-glucan for *Pneumocystis jiroveci* pneumonia, invasive candidiasis, and invasive aspergillosis: systematic review and meta-analysis. J Clin Microbiol 2012; 50:7–15. 22075593 10.1128/JCM.05267-11 PMC3256688

[17] Karageorgopoulos DE, Qu J-M, Korbila IP, Zhu Y-G, Vasileiou VA, Falagas ME. Accuracy of β-D-glucan for the diagnosis of pneumocystis jirovecii pneumonia: a meta-analysis. Clin Microbiol Infect 2013; 19:39–49. 22329494 10.1111/j.1469-0691.2011.03760.x

[18] He S, Hang J-P, Zhang L, Wang F, Zhang D-C, Gong F-H. A systematic review and meta-analysis of diagnostic accuracy of serum 1,3-β-D-glucan for invasive fungal infection: focus on cutoff levels. J Microbiol Immunol Infect 2015; 48:351–61. 25081986 10.1016/j.jmii.2014.06.009

[19] Hou T-Y, Wang S-H, Liang S-X, Jiang W-X, Luo D-D, Huang D-H. The screening performance of serum 1,3-beta-D-glucan in patients with invasive fungal diseases: a meta-analysis of prospective cohort studies. PLoS One 2015; 10:e0131602. 26146829 10.1371/journal.pone.0131602 PMC4493111

[20] Xiaoling L, Tingyu T, Caibao H, Tian Z, Changqin C. Diagnostic efficacy of derum 1,3-β-D-glucan for invasive fungal infection: an update meta-analysis based on 37 case or cohort studies. Open Med (Wars) 2018; 13:329–37. 30211316 10.1515/med-2018-0050 PMC6132083

[21] White SK, Walker BS, Hanson KE, Schmidt RL. Diagnostic accuracy of β-d-glucan (fungitell) testing among patients with hematologic malignancies or solid organ tumors: a systematic review and meta-analysis. Am J Clin Pathol 2019; 151:275–85. 30307463 10.1093/ajcp/aqy135

[22] Rouzé A, Loridant S, Poissy J, et al Biomarker-based strategy for early discontinuation of empirical antifungal treatment in critically ill patients: a randomized controlled trial. Intensive Care Med 2017; 43:1668–77. 28936678 10.1007/s00134-017-4932-8

[23] De Pascale G, Posteraro B, D’Arrigo S, et al (1,3)-β-D-glucan-based empirical antifungal interruption in suspected invasive candidiasis: a randomized trial. Crit Care 2020; 24:550. 32891170 10.1186/s13054-020-03265-y PMC7487510

[24] Shi X, Liu Y, Gu X, et al Diagnostic value of (1 → 3)-β-D-glucan in bronchoalveolar lavage fluid for invasive fungal disease: a meta-analysis. Respir Med 2016; 117:48–53. 27492513 10.1016/j.rmed.2016.05.017

[25] Theel E, Jespersen D, Iqbal S, et al Detection of (1, 3)-β-D-glucan in bronchoalveolar lavage and serum samples collected from immunocompromised hosts. Mycopathologia 2013; 175:33–41. 22945270 10.1007/s11046-012-9579-y

[26] Salerno D, Mushatt D, Myers L, et al Serum and BAL beta-D-glucan for the diagnosis of pneumocystic pneumonia in HIV-positive patients. Respir Med 2014; 108:1688–95. 25448310 10.1016/j.rmed.2014.09.017 PMC4297544

[27] McDonald EG, Afshar A, Assiri B, et al *Pneumocystis jirovecii* pneumonia in people living with HIV: a review. Clin Microbiol Rev 2024; 37:e0010122. 38235979 10.1128/cmr.00101-22 PMC10938896

[28] Fabre V, Markou T, DeMallie K, et al Single academic center experience of unrestricted β-d-glucan implementation. Open Forum Infect Dis 2018; 5:ofy195. 30186888 10.1093/ofid/ofy195 PMC6120669

[29] Ito H, Okamoto K, Yamamoto S, et al Incidence and risk factors for inappropriate use of non-culture-based fungal assays: implication for diagnostic stewardship. Open Forum Infect Dis 2021; 9:ofab601. 35024373 10.1093/ofid/ofab601 PMC8743121

[30] Kritikos A, Poissy J, Croxatto A, Bochud P-Y, Pagani J-L, Lamoth F. Impact of the beta-glucan test on management of intensive care unit patients at risk for invasive candidiasis. J Clin Microbiol 2020; 58:e01996-19. 32238435 10.1128/JCM.01996-19 PMC7269378

