# Supplementary material for: A Diagnostic Stewardship Intervention to Improve Utilization of 1,3 β-D-Glucan Testing at a Single Academic Center: Five-Year Experience
Source: Open Forum Infect Dis. 2024 Jul 1;11(7):ofae358. doi: 10.1093/ofid/ofae358 (PMC11259134; doi:10.1093/ofid/ofae358)
Supplement: ofae358_Supplementary_Data [file ofae358_supplementary_data.docx]

**Supplementary data**

**A. Supplementary figures**

**Figure S1. Monthly numbers of inpatient 1,3-β-D-glucan tests performed in the pre-intervention period and approved inpatient 1,3-β-D-glucan tests in the post-intervention period (per 1,000 inpatient days)**

**Figure S2. Monthly numbers of 1,3-β-D-glucan tests ordered in the pre-intervention period and 1,3-β-D-glucan test requests ordered in the post-intervention period**

**B. Supplementary tables**

**Table S1. The reasons for rejection of the 1,3-β-D-glucan test requests in the post-intervention group**

**Table S2. The specimen types, positivity rate and average turnaround time of 1,3-β-D-glucan tests pre- and post-intervention**

**Table S3. Other diagnostic tests for invasive fungal infections performed in the rejected test group**

**C. Case descriptions of invasive fungal infections in the rejected group**

**A. Supplementary Figures**

Figure S1. Monthly numbers of inpatient 1,3-β-D-glucan tests performed in the pre-intervention period and approved inpatient 1,3-β-D-glucan tests in the post-intervention period (per 1,000 inpatient days)


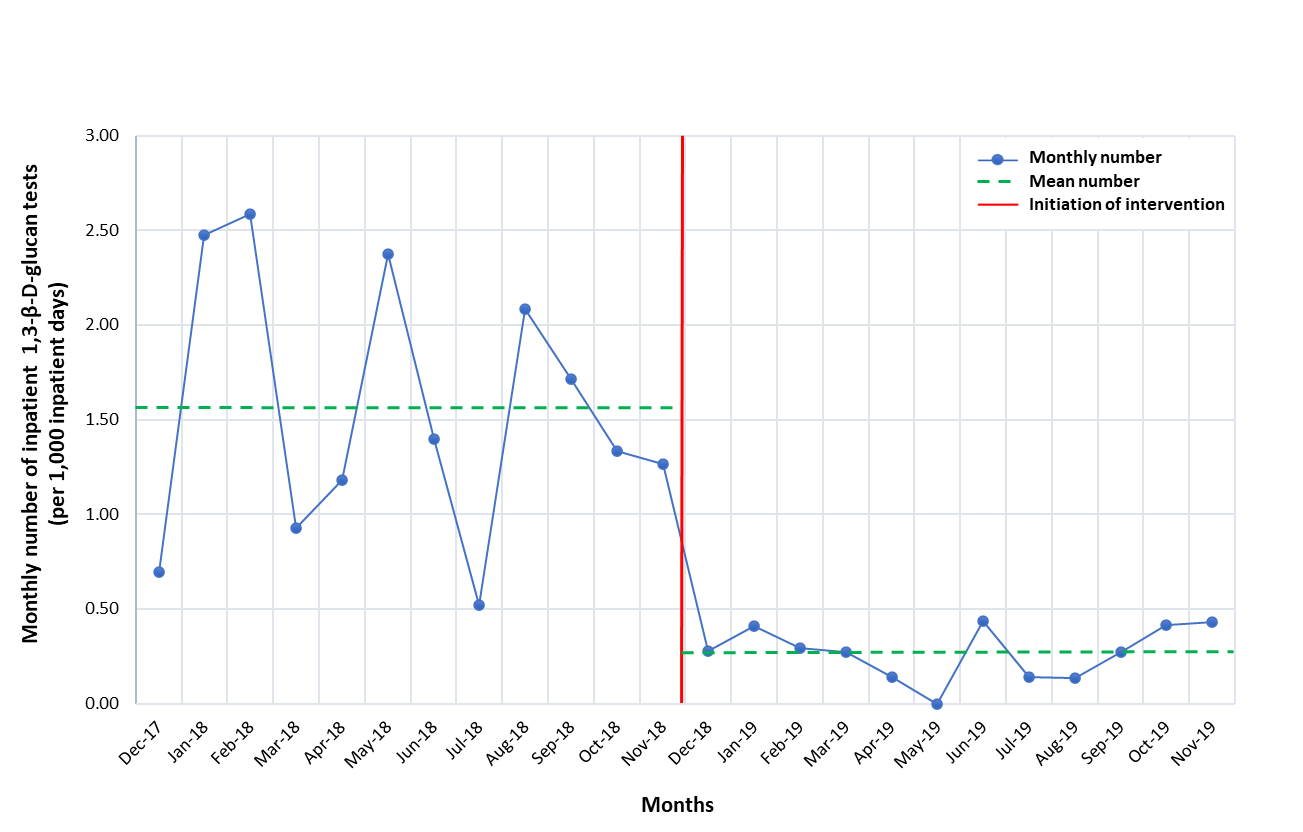


Figure S2. Monthly numbers of 1,3-β-D-glucan tests ordered in the pre-intervention period and

1,3-β-D-glucan test requests ordered in the post-intervention period


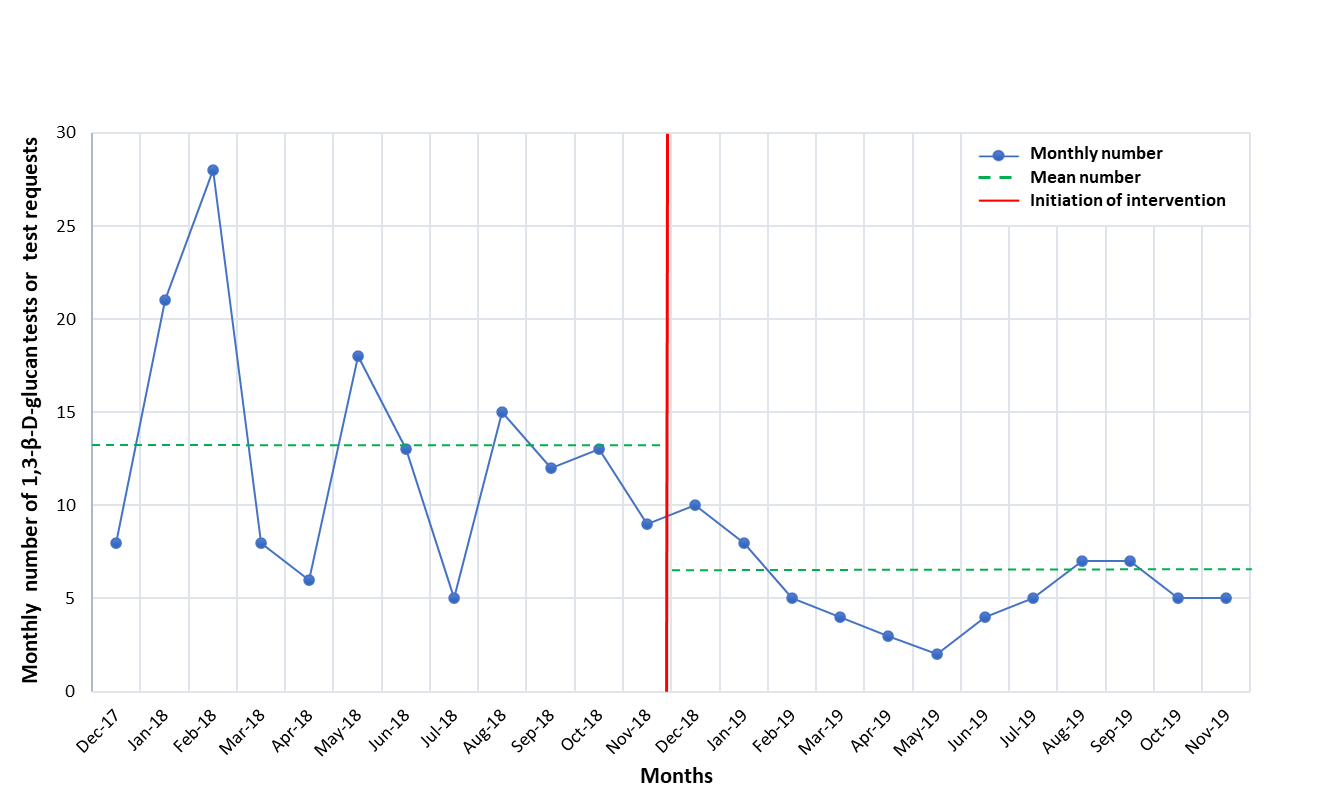


**B. Supplementary Tables**

Table S1. The reasons for rejection of the 1,3-β-D-glucan test requests in the post-intervention group

| **Reasons for BDG test request rejection, n (%)** | **Number of patients**  **N=41** |
| --- | --- |
| Not immunocompromised and not in ICU | 30 (73) |
| Infectious disease service recommended against BDG testing | 5 (12) |
| Uncertain what fungal infection is suspected | 4 (10) |
| Scheduled for bronchoscopy and not on empiric antifungal therapy | 2 (5) |

ICU, intensive care unit; BDG, 1,3-β-D-glucan

Table S2. The specimen types, positivity rate and average turnaround time of 1,3-β-D-glucan tests pre- and post-intervention

|  | **Pre-intervention**  **1,3-β-D-glucan**  **test results**  N=156^a^ | **Post-intervention**  **1,3-β-D-glucan**  **test results**  N=24 | **p-value** |
| --- | --- | --- | --- |
| **Sample types, n (%)** |  |  | **0.043** |
| Serum | 111 (71.2) | 22 (91.7) |  |
| BAL | 45 (28.9) | 2 (8.3) |  |
| **Positive^b^, n (%)** | 39 (25.3) | 11 (45.8) | **0.038** |
| **Turnaround time, mean [SD] hours** | 49.4 [31.1] | 46.9 [30.0] | 0.73 |

BAL, bronchoalveolar lavage

a. One serum specimen and one BAL specimen were resulted as invalid.

b. Positive tests are defined as ≥ 80 pg/mL for serum and > 500 pg/mL for BAL samples.

Table S3. Other diagnostic tests for invasive fungal infections performed in the rejected test group

| **Test name, n (%)** | **Number of patients**  **N=65** |
| --- | --- |
| Fungal culture | 56 (86) |
| BAL cytology GMS stain | 14 (22) |
| Urine *Histoplasma* antigen | 12 (18) |
| BAL *Histoplasma* antigen | 12 (18) |
| Serum galactomannan | 7 (11) |
| Serum *Coccidioides* antibody | 7 (11) |
| Serum *Histoplasma* antibody | 5 (8) |
| Serum *Cryptococcus* antibody | 5 (8) |
| Serum *Blastomyces* antibody | 5 (8) |
| BAL galactomannan | 4 (6) |
| Serum *Histoplasma* antigen | 3 (5) |
| BAL *Coccidioides* antigen | 1 (2) |
| *Pneumocystis jirovecii* PCR | 1 (2) |

BAL, bronchoalveolar lavage; GMS, Grocott’s methenamine silver; PCR, polymerase chain reaction

**C. Case descriptions of invasive fungal infections in the rejected group**

The first case was a patient who was found to have candidemia after abdominal surgery. Blood cultures were collected on the same day as BDG test was requested. The blood cultures became positive in 23 hours with yeast which was subsequently identified as *Candida tropicalis* by multiplex blood culture PCR within 1 hour.

The second case was a patient with HIV/AIDS who was diagnosed with *Talaromyces marneffei* infection in the right tonsil by tissue biopsy. When the BDG test was requested by the primary team, the infectious disease service was already involved in the patient’s care and did not support BDG testing. In addition, the otolaryngology service was consulted next day and agreed to perform biopsy of the tonsillar mass.
